# Interstitial Fibrosis as a Common Counterpart of Histopathological Risk Factors in Papillary Thyroid Microcarcinoma: A Retrospective Analysis

**DOI:** 10.3390/diagnostics14151624

**Published:** 2024-07-27

**Authors:** Can Sahin, Mehmet Arda Inan, Banu Bilezikci, Hasan Bostanci, Ferit Taneri, Ramazan Kozan

**Affiliations:** 1 Department of General Surgery, Yenimahalle Training and Research Hospital, Ankara 06370, Türkiye; 2 Department of Pathology, Gazi University Faculty of Medicine, Ankara 06500, Türkiye; drmardainan@gmail.com; 3 Department of Pathology, Guven Hospital, Ankara 06540, Türkiye; banubil@yahoo.com; 4 Department of General Surgery, Gazi University Faculty of Medicine, Ankara 06500, Türkiye; hasanbostanci@yahoo.com (H.B.); taneriferit@gmail.com (F.T.); dr.kozan@hotmail.com (R.K.)

**Keywords:** interstitial fibrosis, papillary thyroid microcarcinoma, risk factors, thyroid cancer prognosis, papillary cancer variants

## Abstract

Purpose. Interstitial fibrosis in papillary thyroid microcarcinoma is a subject which is under-investigated. The aim of this study is to determine the relationship between interstitial fibrosis, the subtypes of papillary microcarcinoma, and the established prognostic factors. Material and Methods. A total of 75 patients diagnosed with papillary microcarcinoma of the thyroid from January 2011 to December 2020 have been evaluated retrospectively, using demographic features, tumor size, subtype of the tumor, surgical margin status, unifocality, lymphovascular invasion, extracapsular spread and lymph node metastasis as parameters. Hematoxylin and eosin slides were reviewed for interstitial fibrosis. Results. The study includes 13 males and 62 females, in a total of 75 patients. There were 51 patients (68%) with interstitial fibrosis and 24 (32%) patients without interstitial fibrosis. Among them, 45 (60%) were classic, 27 (36%) were follicular variant and 3 (4%) were other subtypes. Interstitial fibrosis is significantly associated with bilaterality (*p* = 0.023), multifocality (*p* = 0.004), capsule invasion (*p* < 0.001) and lymph node metastasis (*p* = 0.043). Evaluation of tumor sub groups showed significant increased risk of lymphovascular invasion in the follicular variant (*p* = 0.019). Conclusion. Although the relationship of interstitial fibrosis and prognosis of other cancer types has been discussed, there are few studies in the literature regarding its effect on the prognosis of papillary microcarcinoma. Our results show that interstitial fibrosis can be used as a risk factor. However, new studies are needed to clearly reveal the physiopathology of interstitial fibrosis and its effect on tumorigenesis.

## 1. Introduction

Papillary thyroid microcarcinoma (PMC) is the definition used for papillary thyroid carcinoma (PTC) with a diameter of 10 mm or less. PMC has a very high 10-year survival rate, of 99% [1]. Recent publications show that PMC has a rate of approximately 30% among all differentiated thyroid carcinomas [2]. The treatment modalities of this disease also involve many controversies such as the adequacy of lobectomy and necessity of prophylactic lymph node dissection [3,4]. PMC is usually asymptomatic, and diagnosed incidentally. Only a very small group of PMCs can be detected on clinical examination; they may present incidentally with neck lymphadenopathies, or may be symptomatic with recurrent nerve palsies [5]. These tumors are found in between 2% and 49.9% of thyroid specimens [3].

There are many subtypes of PMC. Compared to the conventional classic variant, the solid variant, tall-cell variant and diffuse-sclerosing-variant types usually show an aggressive course. The follicular variant, encapsulated variant and Warthin-like variant have a milder course. Previous studies show that aggressive subtypes have lymph node metastasis and extrathyroidal extension more frequently, but overall survival does not change. Similarly, distant metastasis rates are also higher. Some authors have argued that more complex surgeries should be performed in these aggressive types such as the tall-cell variant and diffuse sclerosing variant, that prophylactic central lymph node dissection (CLND) would be beneficial, and that radioactive iodine treatment should be considered [6,7].

In addition to tumor subtype, interstitial fibrosis (IF) is another factor that may influence tumor behavior [8]. IF, characterized by fibroblasts and varying amounts of collagen fibers, has been associated with high recurrence rates and mortality in other cancers, such as lung cancer and hepatic metastasis from colorectal cancer [9,10]. However, there are very few studies on this relationship in PTC [11,12,13]. IF has been observed in various cancer types and may have potential prognostic implications in thyroid papillary cancers. However, the current data do not sufficiently support IF as a definitive poor-prognostic factor in these cancers [12,13]. It may not be included in the guidelines yet, but it is likely to be one of the factors that can be used by physicians to determine prognosis in thyroid papillary cancers in the future.

In this study, we aimed to analyze the effect of IF and tumor subtypes on tumor behavior in PMC. The relationship of IF with well-known poor prognostic factors were examined and we tried to clarify their effects on tumor characteristics.

## 2. Material and Methods

### 2.1. Study Design

A total of 75 patients with papillary microcarcinoma who underwent total thyroidectomy (TT) with CLND, lobectomy + isthmectomy (L + I) with CLND or TT with CLND + LLND at our tertiary institution between 2011 and 2020 were selected.

### 2.2. Data Collection

Data were collected through the Hospital Information Management System (HIMS) and patient files. The specific variables included patient demographics (age, gender), tumor characteristics (size, subtype, focality, and bilaterality), other pathological findings (lymph node metastasis, lymphovascular invasion, extracapsular invasion, perineural invasion, surgical margins, and Hashimoto’s thyroiditis), surgical procedures performed (TT + CLND, L + I + CLND, TT + CLND + LLND), and the presence of IF.

### 2.3. Preoperative Evaluation

All patients underwent detailed thyroid and neck ultrasonography preoperatively. Patients were selected based on specific criteria for undergoing either total thyroidectomy with central lymph node dissection (CLND) or lobectomy + isthmectomy with CLND, as stated in the American Thyroid Association (ATA) guidelines [4]. Lateral lymph node dissection (LLND) was also performed in a small subset of patients presenting with obvious lateral neck metastases. The criteria are as follows:

- Total thyroidectomy with CLND: indicated for patients with multifocal or bilateral tumors, patients with a history of head and neck radiation therapy (RT), a family history of thyroid cancer, or clinical evidence of lymph node metastasis.
- Lobectomy + isthmectomy with CLND: suitable for patients with a single tumor focus less than 1 cm, no extrathyroidal extension, and no clinical lymph node metastasis (clinical N0), provided there is no history of RT, family history of thyroid cancer or an indication to remove the contralateral lobe.

Ultrasonography and suspicious lymph nodes: all patients had preoperative neck ultrasonography to evaluate for suspicious lymph nodes. The criteria used to determine if lymph nodes were suspicious included the presence of microcalcifications, hyperechogenicity, irregular margins, or a round shape. Suspicious lymph nodes identified preoperatively or during surgery prompted further evaluation. Preoperative fine-needle aspiration biopsy (FNAB) and/or thyroglobulin (Tg) washings were performed on sonographically suspicious lymph nodes to confirm metastasis, if these findings would alter the surgical plan. High-risk features of the nodules were assessed according to the American Thyroid Association (ATA) guidelines. These features included extrathyroidal extension, high-grade histology, presence of lymphovascular invasion, extensive nodal involvement, and distant metastases.

### 2.4. Ethical Approval

All procedures performed in this study were in accordance with the ethical standards of the institutional and/or national research committee and the 1964 Helsinki Declaration and its later amendments or comparable ethical standards. This study was approved by The Local Ethical Committee of Gazi University School of Medicine (reference: 02.11.2020/687).

### 2.5. Pathology Review

**Figure 1:**
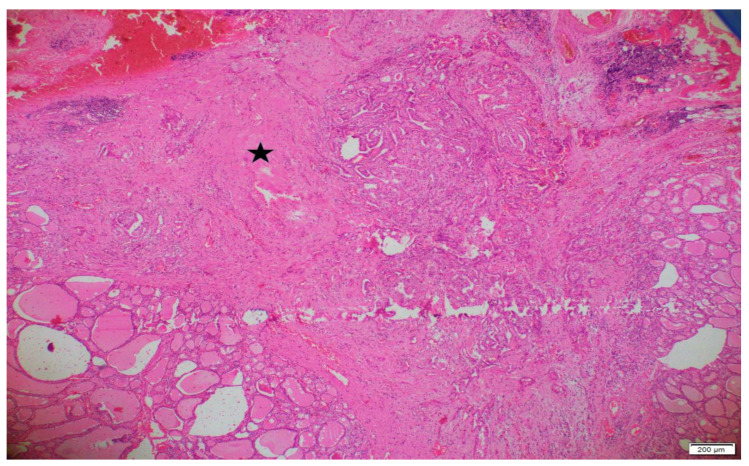
An area of interstitial fibrosis in and around a classic variant papillary microcarcinoma (×40 magnification). The pentagram (*) indicates the region of interstitial fibrosis.

Pathology archive slides of surgical specimens were reviewed by a pathologist, blindly. Patients were divided according to the tumor subtypes, age, gender, diameter of the tumor, capsular invasion, lymphovascular invasion (LVI), perineural invasion, lymph node metastases, multifocality and surgical margins. IF was determined as unorganized collagenous thickening around or in the tumor, which was not relevant, with a capsule formation. An example of IF is represented in Figure 1. While examining IF, hemorrhagic and fibrotic foci due to preoperative FNAB were determined by localization and period of time between interventions, and were excluded.

### 2.6. Inclusion and Exclusion Criteria

The inclusion criteria for the study consisted of several parameters: (1) papillary microcarcinoma diagnosis, (2) at least 3 lymph-node dissections, and (3) surgical procedures performed by the same surgical teams. Exclusion criteria were (1) pediatric patients, (2) inadequate lymph node dissection, and (3) having a secondary carcinoma larger than 1 cm (Table 1).

### 2.7. Statistical Analysis

All statistical analyses were performed with the statistical software SPSS (Statistical Package for Social Sciences) Statistics Version 23.0 software. Before starting the analyses, the conformity of continuous variables to normal distribution was tested using the Kolmogorov–Smirnov Normality Test. Descriptive statistics were presented as mean ± standard deviation and median (minimum–maximum) for continuous variables and frequency (percentage) for categorical variables.

The chi-square test was used as a statistical method. Chi-square test data were explained with the related *p* value and χ^2^ value. The relationships between continuous variables were explained with the Pearson or Spearman correlation coefficient (r) and the related *p* value. The statistical significance value was accepted as *p* < 0.05.

## 3. Results

Of 75 patients, 62 (82.7%) were female and 13 (17.3%) were male. The mean age was 42.56 ±12.84 years. A total of 43 (57%) patients were under the age of 45, while 32 (43%) were older (Table 2).

The postoperative pathological diagnosis of all patients was PMC. According to histological subtypes, there were 45 (60.0%) patients with classic type, 27 (36%) with follicular type, 2 (2.7%) with encapsulated variant, 1 (1.3%) with Warthin-like type. After pathological examination, IF was found in 51 patients (68%). In 24 patients (32%), IF was not detected. Central lymph node dissection (CLND) with total thyroidectomy was performed in 54 (72%), CLND with lobectomy in 12 (16%), and central + lateral neck lymph node dissection with total thyroidectomy in 9 (12%).

The general distribution of prognostic factors was as follows: 28 patients (37.3%) had lymph node metastasis and 47 patients (62.7%) did not. A total of 31 (41.3%) had capsule invasion and 44 (58.7%) did not; 16 (21.3%) had lymphovascular invasion (LVI) and 59 (78.7%) did not. Surgical margins were positive in three (4%) and perineural invasion was detected in two (2.7%). Tumors were unifocal in 48 (64%) and multifocal in 27 (36%). The tumor was bilateral in 20 (26.7%) and unilateral in 55 (73.3%). A total of 34 (45.3%) had chronic lymphocytic thyroiditis.

The mean total size of the tumor, which is the sum of the diameters of all microcancers in a thyroid specimen, was found to be 9.72 ± 6.75 mm and the median value was 8 mm (1–42). The mean of the largest tumor size was 6.64 ± 2.23 mm, with a median of 7 mm (1–10). The mean number of total lymph nodes removed in central or lateral lymph-node dissection was 10.37 ± 11.67, with a median of 6 (3–64). The mean number of metastatic lymph nodes among the removed lymph nodes was 1.44 ± 2.63. The median value was 0 (0–12).

When inferential statistics were performed in our study, since the number of tumor subtypes other than classic and follicular variants was only three (4%), they were excluded. These three patients were excluded only from the subgroup analysis statistics.

According to the results of the statistical analysis, the LVI rate was found to be statistically significantly higher in the follicular type in the comparison between the classic and follicular variants (*p* = 0.019). No significant difference was found in the comparison of other factors (Table 3).

A total of 75 patients were statistically analyzed for IF. The rate of IF was found to be significantly higher in patients with lymph node metastasis (*p* = 0.043). Similarly, IF was shown to be higher in multifocal tumors than in unifocal tumors (*p* = 0.004) (Table 4).

IF is significantly higher in patients with bilateral tumors compared to unilateral tumors (*p* = 0.023). In addition, IF is significantly higher in patients with capsule invasion (*p* < 0.001) (Table 4). Other factors, such as gender, age, tumor size, lymphovascular invasion, extracapsular invasion, perineural invasion, surgical margins and Hashimoto’s thyroiditis were also analyzed. However, there was no statistically significant difference in the presence of IF with these factors (Table 4).

We also analyzed the presence of IF in the classic and follicular variants of PMC. IF was observed in 29 classic cases and 20 follicular cases, while it was absent in 16 classic and 7 follicular cases. A chi-squared test (χ^2^ = 0.345, *p* = 0.557) showed no statistically significant difference between the two variants, suggesting that IF rates are similar in both variants.

## 4. Discussion

This study aimed to investigate the association between interstitial fibrosis (IF) and various prognostic factors in papillary thyroid microcarcinoma (PMC). Our findings suggest that IF is significantly associated with poor prognostic indicators such as lymph node metastasis, multifocality, bilaterality, and capsule invasion. These associations indicate that IF could serve as a valuable marker in assessing the prognosis of PMC.

Prognostic factors in papillary thyroid cancer (PTC) have been reported in various studies in the literature. Several scoring systems also utilize these factors [14,15]. In these studies, female gender, young age, well-differentiated tumor pathology, absence of distant metastasis, tumor size less than 5 cm and absence of extrathyroidal extension were reported as low-risk group characteristics, while male gender, advanced age, poorly differentiated tumor, extrathyroidal extension, tumors larger than 5 cm and presence of distant metastasis were included in the high-risk group and the prognosis in these patients was generally considered to be poor. The results are generally similar in PMC. Age, gender, tumor diameter, LVI, distant metastasis, bilaterality and focality of the tumor have been shown as factors affecting prognosis in some studies in the literature [16,17,18].

The relationship between IF and different cancer types has been shown in some studies in the literature [8,9,10]. However, there are very few studies on the relationship between IF and thyroid malignancies [12,13]. Although some authors have reported that dense fibrosis is an important marker in the diagnosis of PTC [11], the relationship between these two is not clearly understood. The association between IF and tumor behavior in PMC aligns with broader oncological findings regarding fibrosis and malignancy. Our study demonstrates a significant association between IF and poor prognostic factors such as capsule invasion, bilaterality, multifocality, and lymph node metastasis. This correlation is consistent with the role of a stiffened extracellular matrix (ECM) in promoting tumor aggression, as detailed in the literature. Piersma et al. discuss how fibrosis and ECM-stiffening enhance tumor cell growth, survival, and migration through mechanotransduction pathways, ultimately contributing to a more aggressive tumor phenotype [8]. The findings from our study suggest that IF, through similar mechanisms of ECM remodeling and stiffening, may be instrumental in the progression and aggressiveness of PMC. These results underscore the potential of IF as a prognostic marker, supporting its integration into clinical evaluations and treatment planning for PMC. However, further research is necessary to elucidate the precise mechanisms by which IF influences tumorigenesis and to validate its prognostic utility across larger, more diverse patient cohorts.

Cervical lymph node metastasis is also one of the most important prognostic factors. Approximately 75% of PMC recurrences are seen with metastasis in the neck lymph nodes [19]. In many studies, recurrence rate was found to be significantly higher in patients with PMC who had metastatic neck lymph nodes at the time of diagnosis [20,21,22,23]. Similarly, in some studies, cancer-related mortality rates were found to be higher in patients with metastatic lymph nodes [20,23]. The role of CLND in PMC is debated, due to its potential benefits and risks. Proponents argue that CLND improves staging accuracy and reduces locoregional recurrence, particularly in high-risk patients, while opponents highlight the risks of complications such as permanent hypoparathyroidism and recurrent laryngeal nerve injury [24]. Chen et al.’s study demonstrated that prophylactic routine central lymph node dissection (CLND) reduces locoregional recurrence; however, it also increases the incidence of complications such as nerve damage and hypocalcemia [25]. Some guidelines recommend selective CLND based on factors like tumor size, extrathyroidal extension, and aggressive tumor variants. The American Thyroid Association (ATA) guidelines recommend CLND even for T1 tumors, provided that the surgeon is experienced and the associated risk of morbidity is very low. Our study, which included patients who underwent CLND, found that IF was significantly associated with lymph node metastasis, suggesting that patients with IF might benefit from CLND as part of a tailored surgical approach. These findings support the use of individualized treatment plans to balance the benefits of improved staging and recurrence reduction with the risks of surgical complications.

Liu et al. examined the effect of IF on the biological behavior of the tumor in PMC. IF was significantly more common in women. Tumor diameter was found to be higher, and lymph node metastasis was significantly more frequent in patients with IF. Additionally, disease-free survival was higher in patients without IF. Age, extrathyroidal extension and lymph node metastasis were defined as other poor prognostic factors affecting disease-free survival with IF [12]. On the contrary, in the study by Wang et al., survival and prognosis in patients with moderate and advanced IF were found to be significantly higher than patients with low amounts of IF and no IF [13]. Wang et al. suggested that moderate/severe IF could act as a protective factor by potentially encapsulating the tumor and limiting its spread, which might explain the better prognosis observed in these patients.

In our study, unlike Liu’s findings, no significant correlation was found between IF and age or gender when compared with other prognostic factors. Although IF seemed to be higher in women, the difference was not statistically significant (*p* = 0.06). In addition, according to the statistical analysis in our study, no significant correlation was found between tumor diameter and IF.

Fibrosis rate was higher in patients with lymph node metastasis, consistent with previous studies [13]. In addition, statistically significant differences were found with capsular invasion, bilaterality and focality. These associations have not been previously described in the literature, revealing a novel aspect of IF’s prognostic relevance. The relationship between IF and these critical prognostic factors supports the hypothesis that IF contributes to a more aggressive tumor phenotype and poorer prognosis in PMC.

Fibrosis is one of the most specific tissue changes that decreases elasticity, which is a crucial aspect that elastography focuses on [26]. Our study’s findings align with prior research that highlights the utility of ultrasound elastography in thyroid cancer diagnostics. For instance, Petersen et al. demonstrated that the combination of Thyroid Imaging Reporting and Data System (TIRADS) with Shear Wave Elastography (SWE) enhances the accuracy of risk stratification for thyroid nodules, suggesting improved diagnostic performance when these methods are used together [27]. This is consistent with the findings of Leng et al., who explored the role of SWE and connective tissue growth factor (CTGF) in assessing the risk of PTC. They observed that both SWE and CTGF are valuable in evaluating PTC prognosis, with higher elasticity values and CTGF expression correlating with more aggressive tumor behavior [26]. Furthermore, Klarich and White, in their systematic review, highlighted the fact that ultrasound strain elastography (USE) shows promise in managing suspicious thyroid nodules smaller than 10 mm, potentially reducing unnecessary follow-ups and interventions by accurately identifying benign nodules [28]. These insights collectively suggest that integrating advanced imaging techniques such as SWE and USE with traditional ultrasound methods could significantly enhance the diagnostic accuracy and prognostic assessment in thyroid carcinomas, including PMC. The association of IF with adverse prognostic indicators in our study further supports the potential of fibrosis markers as adjuncts in the comprehensive evaluation of thyroid cancer prognosis.

This observation differs from those of other studies on different subtypes of PMC. Zhi et al. found that lymph node metastasis, tumor diameter and extrathyroidal extension were less common in follicular and encapsulated variants compared to the classic variant [29]. Their study reported no significant difference in survival between subtypes. Similarly, Sezer et al. found that the frequency of LVI was significantly different among the histopathological subtypes of PTC, with the lowest LVI rates observed in the follicular variant compared to other subtypes [18]. Altiner et al. found a lower risk of central lymph node metastasis in the follicular variant [30]. Conversely, Conzo et al. showed that locoregional lymph node recurrence is more frequently associated with follicular variant PTC [31]. Discussions regarding the histopathological features of the subtypes continue to evolve, and it is evident that further research is necessary in this field. Due to the small number of patients, we could not analyze subtypes other than the classic and follicular variants. However, our study observed that LVI, a known prognostic factor, was statistically significantly higher in the follicular variant, contrary to some reports in the literature [18,26]. Our study found no significant difference in other factors when comparing subtypes. Additionally, the analysis of IF in the classic and follicular variants of PMC revealed no statistically significant difference between the two variants (*p* = 0.557), indicating that IF rates are similar in both.

Our study has several limitations, including its retrospective design and the relatively small sample size. Additionally, the exclusion of rare PMC variants from our analysis due to small numbers may limit the generalizability of our findings. Future prospective studies with larger cohorts and comprehensive analysis of PMC patients are needed to validate our results and elucidate the underlying mechanisms linking IF to tumor aggressiveness. To enhance clinical utility, future research should validate IF as a prognostic marker and integrate it into refined scoring models, thus providing a more comprehensive risk stratification for PMC patients.

## 5. Conclusions

This study highlights the potential of IF as a risk factor in PMC. In our study, the close relationship of IF with well-known prognostic factors such as capsule invasion, lymph node metastasis and bilaterality, which have been previously proven, indicates that IF itself may be used as a prognostic factor in the future. However, further studies are needed to clearly demonstrate the physiopathology of IF in PMC and its effect on tumorigenesis.

Due to the limited patient population, we could not obtain much data on tumor subtypes in our study. Still, we observed that lymphovascular invasion, a significant prognostic factor, was found more frequently in the follicular type. Future studies should aim to gather a larger patient population to enable a more robust analysis of tumor subtypes and their prognostic implications, thereby providing a clearer understanding of differences among these subtypes.

## Figures and Tables

**Table 1:** Inclusion and exclusion criteria.

Inclusion Criteria	Exclusion Criteria
Papillary microcarcinoma	Patients younger than 18 years old
Dissection of at least 3 lymph nodes	Inadequate lymph node dissection
Same surgical teams	Tumors larger than 1 cm

**Table 2:** Age and gender distribution of the patients.

Age (years)	42.5 ± 12.84 (19–73)
Age groups (<45/≥45)	43/32
Gender (*n*,%)	
- Male	13 (17.3%)
- Female	62 (82.7%)

**Table 3:** Distribution of tumor subtypes according to different demographic and pathological characteristics of patients; χ^2^, chi-square test.

	Classic Variant	Follicular Variant	*p* Value	χ^2^ Value
Gender				
- Male	7 (58.3%)	5 (41.7%)	0.74	0.107
- Female	38 (63.3%)	22 (36.7%)		
Age				
- 18–45	27 (64.3%)	15 (35.7%)	0.71	0.137
- 45+	18 (60%)	12 (40%)		
Tumor size				
- 0–5 mm	14 (63.6%)	8 (36.4%)	0.895	0.17
- Over 5 mm	31 (62%)	19 (38%)		
Lymph node metastasis				
- Yes	17 (63%)	10 (37%)	0.95	0.004
- No	28 (62.2%)	17 (37.8%)		
Focality				
- Multifocal	13 (50%)	13 (50%)	0.139	2.71
- Unifocal	32 (69.6%)	14 (30.4%)		
Bilaterality				
- Bilateral	9 (47.4%)	10 (52.6%)	0.112	2.522
- Unilateral	36 (67.9%)	17 (32.1%)		
Lymphovascular invasion				
- Yes	6 (37.5%)	10 (62.5%)	0.019	5.486
- No	39 (69.6%)	17 (30.4%)		
Extracapsular invasion				
- Yes	15 (50%)	15 (50%)	0.064	3.429
- No	30 (71.4%)	12 (28.6%)		
Perineural invasion				
- Yes	0 (0%)	2 (100%)	0.064	3.429
- No	45 (64.3%)	25 (35.7%)		
Surgical margin				
- Positive	2 (66.7%)	1 (33.3%)	0.879	0.023
- Negative	43 (62.3%)	26 (37.7%)		
Hashimoto’s thyroiditis				
- Yes	23 (67.6%)	11 (32.4%)	0.393	0.728
- No	22 (57.9%)	16 (42.1%)		

**Table 4:** Distribution of tumor interstitial-fibrosis status according to different demographic and pathological characteristics of patients; χ^2^, chi- square test.

	Interstitial Fibrosis		*p* Value	χ^2^ Value
	Yes	No		
Gender				
- Male	6 (46.2%)	7 (53.8%)	0.063	3.449
- Female	45 (72.6%)	17 (27.4%)		
Age				
- 18–45	33 (76.7%)	10 (23.3%)	0.06	3.541
- 45+	18 (56.3%)	14 (43.7%)		
Tumor size				
- 0–5 mm	13 (59.1%)	9 (40.9%)	0.287	1.136
- Over 5 mm	38 (71.7%)	15 (28.3%)		
Lymph node metastasis				
- Yes	23 (82.1%)	5 (17.9%)	0.043	4.107
- No	28 (59.6%)	19 (40.4%)		
Focality				
- Multifocal	24 (88.9%)	3 (11.1%)	0.004	8.46
- Unifocal	27 (56.2%)	21 (43.8%)		
Bilaterality				
- Bilateral	18 (90%)	2 (10%)	0.023	6.066
- Unilateral	33 (60%)	22 (40%)		
Lymphovascular invasion				
- Yes	13 (81.3%)	3 (18.8%)	0.2	1.641
- No	38 (64.4%)	21 (35.6%)		
Extracapsular invasion				
- Yes	29 (93.5%)	2 (6.5%)	<0.001	15.85
- No	22 (50%)	22 (50%)		
Perineural invasion				
- Yes	2 (100%)	0 (0%)	0.325	3.449
- No	49 (67.1%)	24 (32.9%)		
Surgical margin				
- Positive	1 (33.3%)	2 (66.7%)	0.189	1.726
- Negative	50 (69.4%)	22 (30.6%)		
Hashimoto’s thyroiditis				
- Yes	21 (61.8%)	13 (38.2%)	0.292	1
- No	30(73.2%)	11 (26.8%)		

## Data Availability

The raw data supporting the conclusions of this article will be made available by the authors on request.
